# Molecular Characterization of Feline Leukemia Virus from Cats in Shelters in the Metropolitan Region of Recife, Pernambuco, Northeast Brazil

**DOI:** 10.1007/s00284-026-05191-8

**Published:** 2026-10-23

**Authors:** Cláudia Kathariny da Silva Farias, Phelipe Magalhães Duarte, Wilson Jose da Silva Junior, Caio Andrey Bezerra Januário, Rita de Cássia Carvalho Maia, Otávio Valério de Carvalho, José Wilton Pinheiro Junior

**Affiliations:** 1 Independent Veterinary Researcher, Recife, Brazil; 2 Postgraduate Program in Animal Bioscience, Federal Rural University of Pernambuco (UFRPE), Dom Manuel de Medeiros Street, n/a - Dois Irmãos, Recife, Pernambuco 52171-900 Brazil; 3 Department of Genetics and Molecular Biology, Federal University of Pernambuco (UFPE), Prof. Moraes Rego avenue, 1235 - Cidade Universitária, Recife, Pernambuco 50670-901 Brazil; 4 Department of Veterinary Medicine, Federal Rural University of Pernambuco (UFRPE), Dom Manuel de Medeiros Street, n/a - Dois Irmãos, Recife, Pernambuco 52171-900 Brazil; 5 Tecsa Laboratories, do Contorno avenue, 6226 - Funcionários, Belo Horizonte, Minas Gerais Brazil

## Abstract

This research aimed to conduct an epidemiological-molecular study of feline leukemia virus (FeLV) infection in cats from shelters in the Metropolitan Region of Recife (MRR), Pernambuco, Northeast Brazil. Blood samples from 260 cats from 18 shelters located in 5 municipalities within the MRR were analyzed. DNA samples from peripheral blood were subjected to Polymerase Chain Reaction (PCR) to detect FeLV proviral DNA. PCR-positive samples were sequenced and analyzed using BLAST software for phylogenetic tree construction. The prevalence of infection in cats was 9.6% (25/260). Among the 18 sampled shelters, 33.3% (6/18) had cats positive for FeLV. It was observed that 60% (3/5) of the investigated municipalities had felines positive for FeLV infection. Among the positive cats, males accounted for 52% (13/25) and females for 48% (12/25). Of the 22 sequenced samples, 18 sequences were used as queries for an online BLAST search to build a dataset. This study is pioneering in the state of Pernambuco and demonstrates the prevalence of FeLV infection in felines living in shelters in the Metropolitan Region of Recife, PE. Based on these results, we recommend vaccinating any cat with outdoor access in the study region. Understanding the prevalence of FeLV in Pernambuco provides insights into the local situation and helps develop effective hygienic-sanitary programs in feline shelters in the MRR, contributing to increased awareness of responsible pet ownership and reducing FeLV transmission among community felines.

## Introduction

Feline leukemia virus (FeLV) is a cosmopolitan retrovirus belonging to the genus *Gammaretrovirus* and the species *Gammaretrovirus felleu* [1], associated with multifaceted pathogenesis including hematological, immunosuppressive, and oncological changes [2], significantly impacting the lifespan of domestic and wild felids [3]. Transmission primarily occurs through direct contact via saliva and bodily fluids such as milk, urine, and feces, and through blood transfusions [3]. The virus can also be transmitted through bites, grooming, or shared fomites, especially among group cats [4].

Cat colonies in shelters may increase the risk of infectious diseases due to continuous contact and high infection pressure [5]. Shelters are crucial as temporary homes for stray animals, protecting them for subsequent rehabilitation and reintegration into society through responsible adoption [6].

FeLV is primarily subdivided into four subgroups: FeLV-A, which is dominant, less pathogenic, and less virulent, causing immunosuppression; FeLV-B, resulting from recombinations between subgroup A and enFeLV, associated with hematopoietic neoplasms; FeLV-C, originating from recombinations or mutations of subgroup A, related to non-regenerative anemias; and FeLV-T, with a cytolytic effect on T lymphocytes, resulting in severe immunosuppression [4]. Subgroups resulting from mutations related to recombinations of envelope protein genes are more pathogenic than FeLV-A [7].

Prevalence data for FeLV have been reported in various regions: North America [8], South America [9], Asia [3], Africa [10], Oceania [11].

, and Europe [12]. However, there are few studies on the prevalence of viral subgroups [7].

In Brazil, studies on FeLV prevalence have shown greater variability compared to other countries, with research conducted in only a few Brazilian states [7, 13]. Two Brazilian studies characterized the circulating subgroups, differentiating FeLV-A and FeLV-B in Santa Catarina [14] and FeLV-A in Maranhão [7].

This study aimed to identify the prevalence of feline leukemia virus through the detection of proviral DNA and perform molecular characterization of FeLV-positive samples, determining the circulating viral subgroup and phylogenetic relationships in felines from the Metropolitan Region of Recife, Pernambuco, Brazil.

## Materials and Methods

### Study Area

**Fig. 1:**
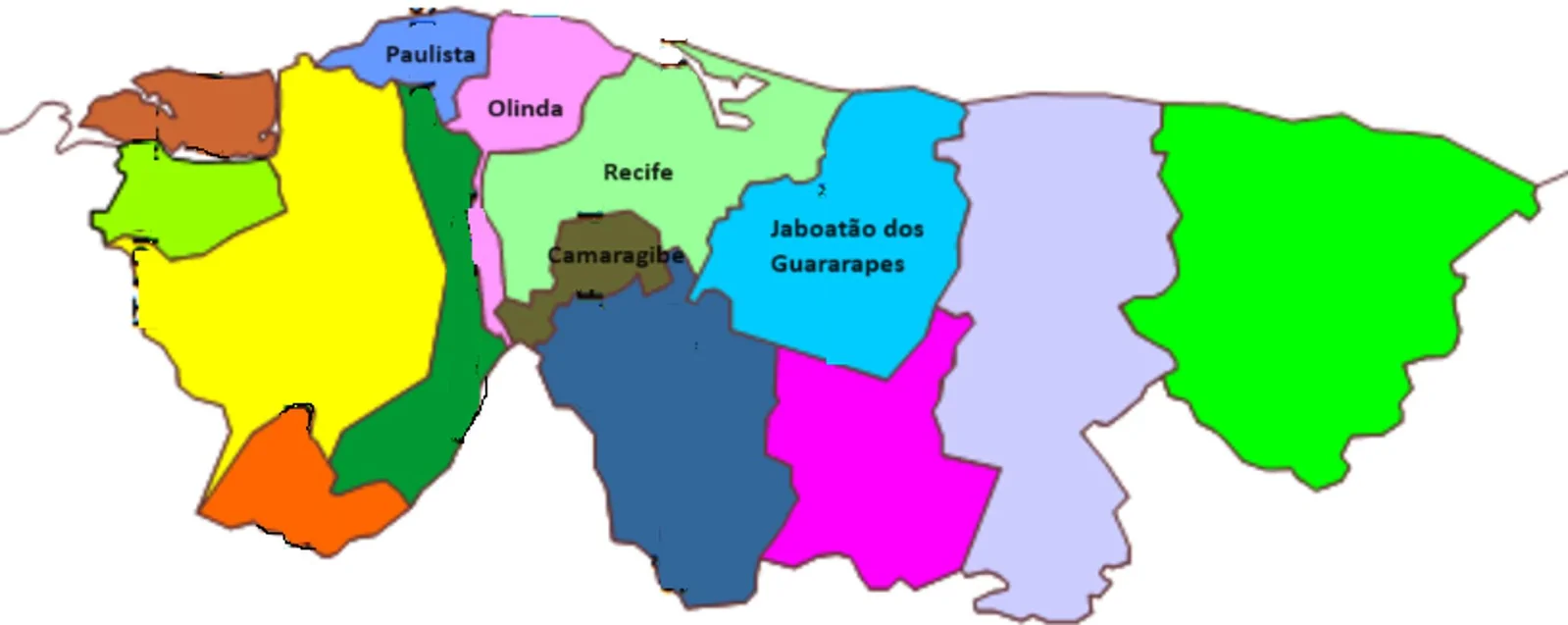
Map of the Metropolitan Region of Recife with identification of the sampling cities. Adapted from Silva Filho and Raia Junior [16].

This study was conducted in shelters housing cats in the municipalities of Recife, Olinda, Paulista, Camaragibe, and Jaboatão dos Guararapes, located in the Metropolitan Region of Recife (RMR/PE), which comprises 15 municipalities [15] and is situated in the state of Pernambuco, Northeast Brazil. Sampling was conducted in 5 of the 15 municipalities (Recife, Olinda, Paulista, Camaragibe, Jaboatão dos Guararapes) (Fig. 1).

### Sampling

To determine the sample size for the prevalence study, a total of 1,570,929 cats was considered [17]. Using an average prevalence interval of 16.7% [18], a minimum confidence level of 95%, and a statistical error of 5%, the sample size (n) examined was 260 cats. Collections were conducted from March 2022 to July 2023, adhering to the following inclusion criteria: unvaccinated felines and those over six months of age. Sex, breed, origin, and clinical condition were not exclusion factors.

### Biological Sample Collection

Approximately 3 mL of whole blood samples were collected by cephalic or jugular venipuncture, after prior antisepsis with a 70% ethanol solution. The biological material was stored in an EDTA tube, properly labeled, and transported refrigerated to the Laboratory of Domestic Animal Viruses (LAVIAN - UFRPE). The samples were then stored at -20 °C until processing.

### DNA Extraction and PCR

The 260 DNA samples from peripheral blood were subjected to PCR for the detection of FeLV proviral DNA. DNA extraction from whole blood was performed using a commercial kit (Wizard^®^ Genomic DNA Purification Kit - A1125, Promega, USA), following the manufacturer’s recommendations.

Amplification was carried out by conventional PCR using primers based on the gag sequence, which is common to all subtypes of exogenous FeLV, producing a 450 bp fragment (3′-ATGGCTGTCCCACTAGAG-5′ (reverse) and 5′-ACTAACCAATCCCCACGC-3′ (forward) [19]. The final volume of the PCR reaction was 25 µL, consisting of 12.5 µL of PCR Mix (containing Taq DNA polymerase, dNTPs, MgCl2, and PCR buffer), 1.25 µL of reverse primer, 1.25 µL of forward primer, 2 µL of sample DNA, and 8 µL of nuclease-free water.

The thermal cycler protocol included the following steps: an initial denaturation cycle at 94 °C for 5 min, followed by 35 cycles of 94 °C for 1 min, 54 °C for 1 min, 72 °C for 1 min, and a final extension at 72 °C for 5 min. Electrophoresis was performed on a 1% agarose gel [19]. Controls were kindly provided by Tecsa Laboratories.

### DNA Purification and Sequencing

Purification of the PCR-amplified products was carried out using the commercial ReliaPrep™ DNA Clean-Up and Concentration System (A2892, Promega, USA), following the manufacturer’s recommendations.

### Sequencing and Phylogenetic Analysis

The samples were sequenced at the UFPE Genetics Laboratory. A total of 22 partial gag gene sequences were sequenced; however, 4 samples were removed due to lack of consensus. The remaining 18 sequences were used as queries in a BLAST online search to assemble a dataset containing the best hits from the non-redundant (nr) protein database.

For this analysis, alignment was filtered using Gblocks v.0.91b. The K2P+G4 model was applied through ModelFinder, and implemented in IQtree. The chosen software for implementing and executing Bayesian methods was Mr Bayes v.3.2.7a. The software was run on the CIPRES Science Gateway, which provides advanced computational resources for phylogenetic and evolutionary analyses.

For parameter inference, the Monte Carlo Markov Chain (MCMC) technique was used. The MCMC configuration was set as follows: number of generations: 50,000,000; burn-in period: 1,250,000 generations (25% of the total); and thinning interval: 1,000 (after the burn-in period, one sample every 1,000 generations was considered to reduce autocorrelation between samples).

MCMC chain convergence was assessed using standard diagnostics with the Tracer software, including the Gelman-Rubin statistic to check convergence between multiple independent chains, autocorrelation to ensure sample independence, and visual inspection of chain trajectories for mixing and stationarity. After verifying convergence, the samples obtained from the posterior distributions were used to estimate parameters of interest, calculating the mean, median, 95% credibility interval, and other relevant statistics. Additionally, predictions based on the posterior distributions of the parameters were generated and compared with true or expected data to validate the model and the effectiveness of the inference method used.

## Results and Discussion

### Prevalence of FeLV Infection

The prevalence of feline leukemia virus (FeLV) infection in the Metropolitan Region of Recife, Pernambuco, Northeast Brazil was 9.5% (25/260).

The felines were evaluated in 18 shelters across 18 different neighborhoods in the Metropolitan Region of Recife (Table 1), where they lived in colonies and had unknown histories and origins.

Among the positive felines, males accounted for 52% (13/25), compared to 48% (12/25) for females. Regarding the municipalities, it was observed that 60% (3/5) had cats positive for FeLV infection.

**Table 1:** Geographical distribution of feline shelters, number of tested felines, and quantity of positive felines

CITY	PREVALENCE IN THE MUNICIPALITY	NEIGHBORHOOD	SHELTER POPULATION	TESTED	FeLV POSITIVES
Olinda	12 (40,0%)	Rio Doce	17	4	− (0,0%)
		Passarinho	58	30	12 (40,0%)
Recife	8 (32,0%)	Encruzilhada	87	28	2 (7,1%)
		Vasco da Gama	12	14	1 (7,1%)
		Monteiro	100	12	− (0,0%)
		Imbiribeira	10	5	− (0,0%)
		Engenho do Meio	15	10	− (0,0%)
		Sítio dos Pintos	80	14	3 (21,4%)
		Estância	20	15	− (0,0%)
		Cajueiro	60	24	− (0,0%)
		Guabiraba	11	8	2 (25,0%)
		Macaxeira	15	12	− (0,0%)
		Passarinho	18	13	− (0,0%)
Paulista	5 (45,4%)	Janga	15	11	5 (45,4%)
		Maranguape	21	14	− (0,0%)
Jaboatão dos Guararapes	− (0,0%)	Cajueiro Seco	40	5	− (0,0%)
		Jordão	10	6	− (0,0%)
Camaragibe	− (0,0%)	Aldeia	38	35	− (0,0%)
**TOTAL TESTED**				**260**	**25 (9**,**6%)**

### Phylogenetic Analysis

**Fig. 2:**
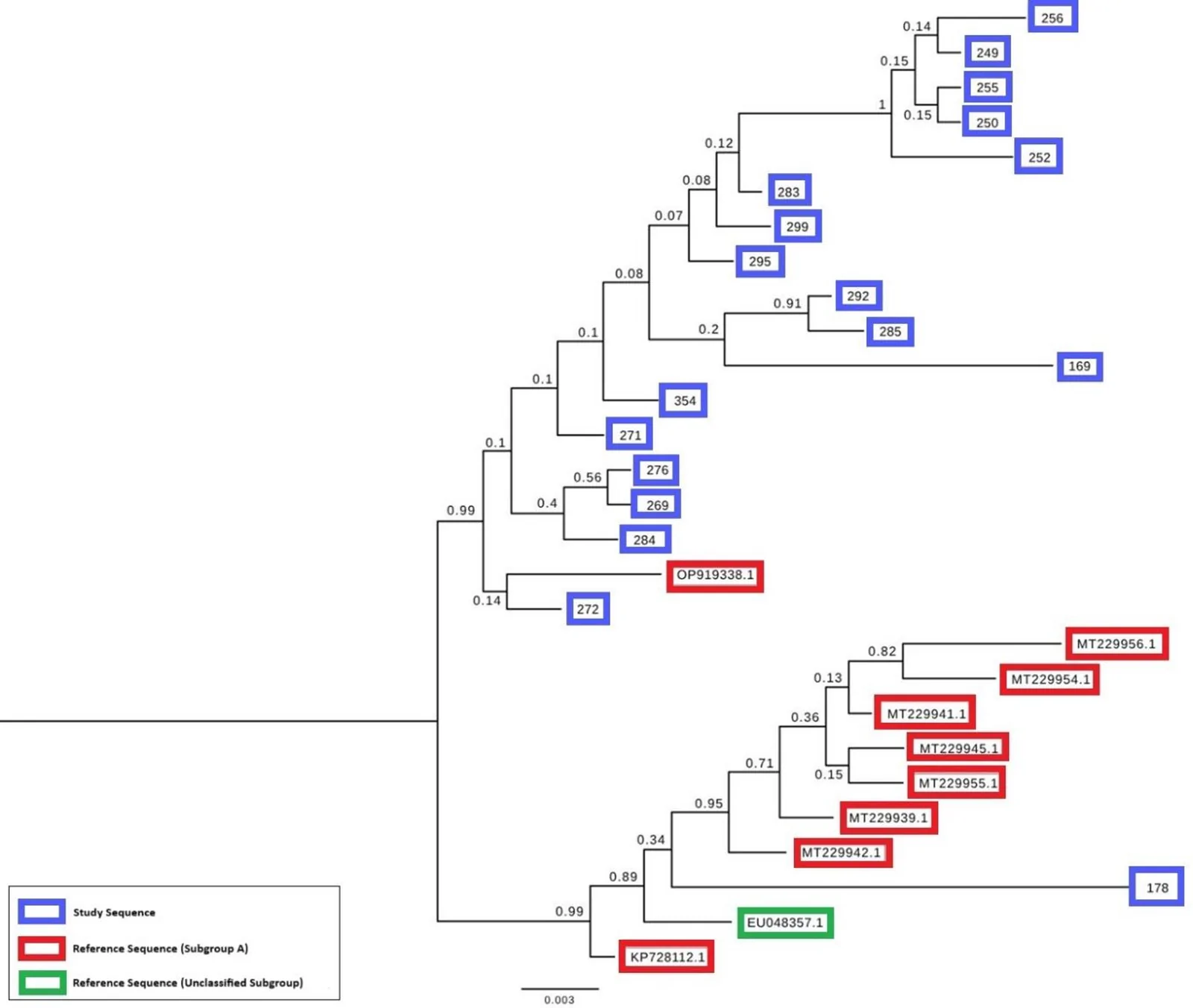
Phylogenetic tree based on the gag gene of the amplified sequences showing 2 major identified groups. Bootstrap values are provided at the nodes of the tree. The subgroup A reference sequence (red) and the unclassified reference sequence (green) were obtained from the National Center for Biotechnology Information (NCBI) for the construction of the database

Phylogenetic analyses of the FeLV-positive samples were based on the amplification of the gag gene of the virus to construct the phylogenetic tree (Fig. 2**)**.

Two major groups were identified with a 99% likelihood of the feline leukemia virus. The primary clade contains 17 sequences from the study, including 1 reference sequence corresponding to the FeLV-A subgroup. The second major clade includes 1 sequence from the study with 9 reference sequences identified, of which 8 correspond to the FeLV-A subgroup and 1 is an unclassified FeLV. The reference sequences are described in Table 2. All obtained sequences were deposited in GenBank, with accession numbers compiled in Table 3.

**Table 2:** Reference sequences obtained from the National Center for Biotechnology Information (NCBI) for database construction

SEQUENCE	SUBGROUP	COUNTRY	REFERENCE
OP919338.1	A	Brazil	Biezus et al. [20]
MT229956.1	A	Colombia	Ortega et al. [9]
MT229954.1	A	Colombia	Ortega et al. [9]
MT229941.1	A	Colombia	Ortega et al. [9]
MT229945.1	A	Colombia	Ortega et al. [9]
MT229955.1	A	Colombia	Ortega et al. [9]
MT229939.1	A	Colombia	Ortega et al. [9]
MT229942.1	A	Colombia	Ortega et al. [9]
EU048357.1	-	Brazil	Coelho et al. [21]
KP728112.1	A	Scotland	Helfer-Hungerbuehler et al. [22]

**Table 3:** List of blood samples from cats in Pernambuco, Brazil, sequenced and deposited in the NCBI

IDENTIFICATION	ABBREVIATION	GENBANK
LAVIAN_FeLV_178	178	QB018110
LAVIAN_FeLV_272	272	QB018111
LAVIAN_FeLV_284	284	QB018112
LAVIAN_FeLV_269	269	QB018113
LAVIAN_FeLV_276	276	QB018114
LAVIAN_FeLV_271	271	QB018115
LAVIAN_FeLV_354	354	QB018116
LAVIAN_FeLV_169	169	QB018117
LAVIAN_FeLV_285	285	QB018118
LAVIAN_FeLV_292	292	QB018119
LAVIAN_FeLV_295	295	QB018120
LAVIAN_FeLV_299	299	QB018121
LAVIAN_FeLV_283	283	QB018122
LAVIAN_FeLV_252	252	QB018123
LAVIAN_FeLV_250	250	QB018124
LAVIAN_FeLV_255	255	QB018125
LAVIAN_FeLV_249	249	QB018126
LAVIAN_FeLV_256	256	QB018127

This study on the epidemiology of Feline Leukemia Virus (FeLV) infection was pioneering in the state of Pernambuco, Brazil, by determining and categorizing the presence of the virus in the region through a phylogenetic perspective. The study alerts the public and veterinarians about the current status of this disease in feline medicine and shelter medicine.

The prevalence of FeLV in Pernambuco was higher than that found in studies from Bahia, Maranhão, and Ceará, states in the northeastern region of Brazil, with prevalences of 3%, 5%, and 5.8%, respectively [7, 23, 24]. Prevalence data in Brazil ranges from 0.3% to 78.4% [7, 18, 20, 21, 25]. This variation may result from differences in sampling, diagnostic techniques, and the health status of the selected animals. For example, Rocha et al. [24] used a sample of 138 cats with immunochromatography for identifying positive animals, whereas Bahia and Maranhão used PCR with 230 and 88 cats tested, respectively.

Similar to the present study, Martins et al. [7] reported prevalences of 5%, and conducted research using similar methodologies with only random samples from shelters. Despite using different sampling methods, they presented prevalences close to those found in studies with pet cats, which is consistent with literature indicating that FeLV is expected to be active and circulating in cat colonies [21]. The prevalences found in studies conducted in feline shelters may result from close contact among colony members, due to the facilitated transmission of retroviruses in high social proximity rates [26].

Cat colonies may promote retrovirus infections due to frequent contact and facilitated transmission of these agents [24], as observed in other studies conducted in high-density environments [7]. In studies with sick animals, FeLV prevalence is usually higher than in studies with clinically healthy animals selected randomly [21, 23].

Studies conducted in North America (3.1%) [8], Oceania (1%) [11], and Europe (3.4%) [27] showed lower prevalences than in Pernambuco. These differences are attributed to varying geographic regions, pet owner education levels, vaccination practices [27], economic power, as well as different management practices, diagnostic techniques, and sampling population density disparities [9].

Vaccines are important allies in controlling viral spread. In many parts of the world, diseases related to the feline leukemia virus (FeLV) are endemic, including in Brazil [28]. In these locations, the FeLV vaccine is classified by the WSAVA [29] as essential for young cats (under 1 year of age) and for adult cats with outdoor access or those living with other cats that have outdoor access.

Preventive measures such as vaccination, sterilization, and increased frequency of rapid diagnostic tests are used in some countries to reduce prevalence [27]. In Brazil, these measures are being implemented slowly due to the country’s diverse socioeconomic conditions [23].

Regarding sex, among positive felines, males accounted for 52% (13/25) and females for 48% (12/25). However, this result should be interpreted with caution, given that the present study was conducted in community catteries where information regarding the origin and history of each animal was unavailable.

Unlike the study by Zanutto et al. [30], the population density in shelters did not influence the prevalence in this study, which aligns with Gleich et al. [27], where population size did not affect the identified prevalence. Sanitary management and infection pressure are additional factors that may influence local prevalence [5].

The described methodology allowed for a robust and detailed analysis through Bayesian inference, ensuring accurate estimates for the model parameters and a thorough understanding of the posterior distribution. The approach used, with a high number of generations, ensured convergence and the quality of the obtained samples, allowing for a reliable and consistent analysis of the results. The use of MrBayes on the CIPRES platform was crucial for the efficiency and success of the analysis, providing the computational resources needed to process the large amount of generated data.

All infected cats will harbor FeLV-A, as this is the transmissible subtype [9, 14]. In contrast, only a few infected cats will have another subtype, such as FeLV-B [14]. However, in this study, where diversity of subgroups was expected due to the seasonal passage of animals in shelters, sample 178 (Fig. 2) showed 95% similarity with reference MT229942.1 identified in Colombia [9], suggesting that this study sequence belongs to the same grouping as the reference sequence. Conversely, study sequences showing various similarities but not deposited in the genetic bank may suggest speciation.

Prevalence studies on FeLV are increasingly necessary in countries without widespread vaccination to understand the global dissemination of FeLV, aiming to contain the virus through vaccination and identification of risk factors associated with this infection. Growing global awareness has led to more frequent routine rapid tests, resulting in the identification of FeLV-positive cats and facilitating veterinarians’ decisions on the medical care needed for these animals. This was the first investigation into FeLV prevalence and phylogenetic relationships in Pernambuco, Northeast Brazil, confirming the circulation of the virus in the study region.

## Conclusion

This study is pioneering in Pernambuco and demonstrates the predominant identification of FeLV-A subgroup and the suggestion of speciation of FeLV variants, as well as the prevalence of FeLV infection in felines living in shelters in the Metropolitan Region of Recife-PE. It reaffirms the importance of testing to identify infected cats. The collection of prevalence data on FeLV provides support for veterinary planning of therapeutic and vaccination programs in feline shelters, contributing to the subsequent reduction of the virus transmission among community felines.

## Data Availability

The Feline leukemia virus (FeLV) sequences obtained in this study were submitted to the National Center for Biotechnology Information (NCBI) and are publicly accessible under accession numbers QB018110 to QB018127.
